# Synthesis, Crystal Structure, Absolute Configuration and Antitumor Activity of the Enantiomers of 5-Bromo-2-chloro-*N*-(1-phenylethyl)pyridine-3-sulfonamide

**DOI:** 10.3390/molecules201119740

**Published:** 2015-11-24

**Authors:** Zhixu Zhou, Linwei Li, Ning Yan, Lei Du, Changshan Sun, Tiemin Sun

**Affiliations:** 1 Key Laboratory of Structure-Based Drug Design and Discovery, Shenyang Pharmaceutical University, Ministry of Education, Shenyang 110016, China; niuniuzzx@163.com (Z.Z.); xt_llw@163.com (L.L.); ningyan@163.com (N.Y.); dl_pharma@163.com (L.D.); 2 Pharmacy Department, Shenyang Pharmaceutical University, Shenyang 110016, China

**Keywords:** pyridinesulfonamide, enantiomer, X-ray diffraction, absolute configuration, DFT, ECD, PI3K, antitumor activity

## Abstract

Pyridinesulfonamide is an important fragment which has a wide range of applications in novel drugs. *R*- and *S*-isomers of 5-bromo-2-chloro-*N*-(1-phenylethyl)pyridine-3-sulfonamide have been synthesized, and the stereostructures have been researched. Single crystals of both compounds were obtained for X-ray analysis, and the absolute configurations (ACs) have been further confirmed by electronic circular dichroism (ECD), optical rotation (OR) and quantum chemical calculations. The crystal structures and calculated geometries were extremely similar, which permitted a comparison of the relative reliabilities of ACs obtained by ECD analyses and theoretical simulation. In addition, the effect of stereochemistry on the PI3Kα kinase and anticancer activity were investigated. Compounds **10a** and **10b** inhibit the activity of PI3Kα kinase with IC_50_ values of 1.08 and 2.69 μM, respectively. Furthermore, molecular docking was performed to analyze the binding modes of *R*- and *S*-isomers.

## 1. Introduction

Sulfonamides were the first clinically available antibacterial agents, and they have been widely used in the design of drug candidates. Sulfonamides have been found to possess a large number of different biological activities, including antibacterial, antiviral, antidiabetic, diuretic, antitumor, and antithyroid activities [1,2]. Recently, pyridine-3-sulfonamide derivatives have been selected as phosphatidylinositol 4-kinase (PI4K) inhibitors and phosphoinositide 3-kinase (PI3K) inhibitors [3,4,5,6,7,8,9]. Another potential use for sulfonamide compounds is (stereoselective) organocatalysis due to their capacity for forming fairly weak hydrogen bonds and increasing acidity compared to amide group [10].

**Figure 1:**
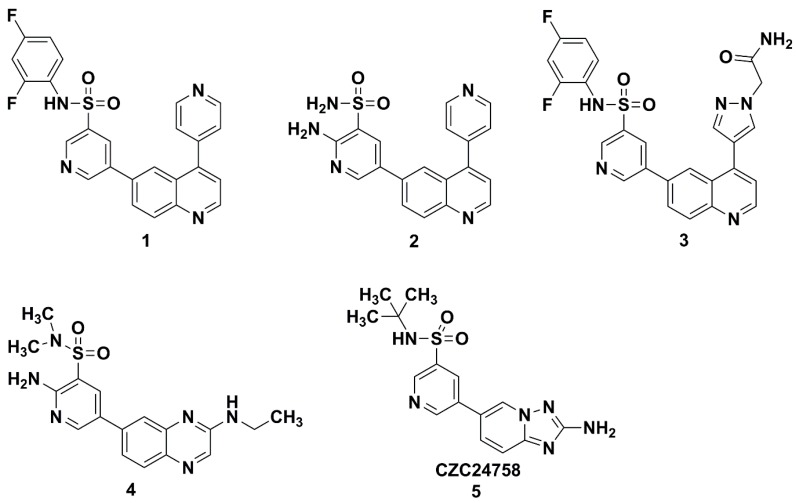
Structures of pyridine-3-sulfonamide derivatives of PI3K inhibitor.

**Figure 2:**
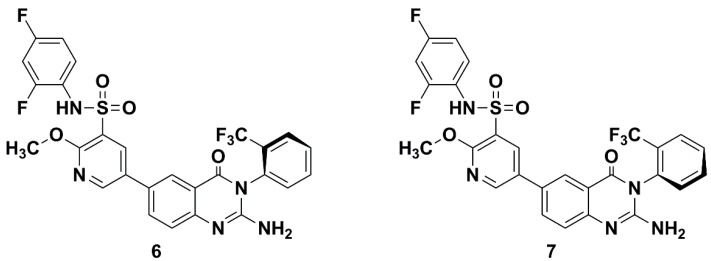
Structure of pyridine-3-sulfonamide derivatives of PI4K inhibitor.

Pyridine-3-sulfonamide derivatives have a wide range of applications in novel antineoplastic and anti-hepatitis C drugs [5,6,7,8,9]. Compounds **1**, **2**, **3** and **4** (Figure 1) are pyridine-3-sulfonamide derivatives with nanomolar activity against the PI3K; these compounds inhibit the activity of PI3Kα with IC_50_ of 1 nM, 10 nM, 7.9 nM and 8 nM, respectively [7,11,12]. CZC24758 (**5**) (Figure 1) is a potent, orally bioavailable small-molecule inhibitor of PI3K [4]. Compounds **6** and **7** (Figure 2) are a pair of enantiomers of chiral pyridine-3-sulfonamide derivatives which display powerful potential for inhibiting hepatitis C virus (HCV). Compound **6** is the first extremely selective PI4K IIIα inhibitor, and exhibiting 25 × higher PI4K IIIα potency than compound **7** (pIC_50_ of 8.3 *vs.* 6.9, pIC_50_ = −log IC_50_) [3]. The pyridine-3-sulfonamide moiety has strong bioactivity because it can act as a hydrogen bond receptor and donor; many studies on chemical modification have shown the pyridine-3-sulfonamide moiety is essential for the activity.

Chirality significantly influences the biological and pharmacological properties of a drug [13]. The stereoisomeric composition of drug substances has become a critical issue in the development, approval and clinical use of drugs. Furthermore, stereoselectivity in drug action and disposition has become a well-recognized consideration in clinical pharmacology and development of chiral drugs. Therefore, it is an urgent problem to determine the absolute configuration of compounds. Nowadays, many techniques are available to determine the absolute configuration of chiral molecules. X-ray diffraction (XRD) has played and continues to play an important role in determining the absolute configuration (AC) of a chiral molecule which must be a single crystal of this molecule. As not every molecule can obtain high quality crystals and due to the limitation of XRD’s application range, optical rotation (OR) is an ideal alternative for the AC of a molecule, which is based on enantiomers of chiral molecules exhibiting specific rotations at frequency *v*, [a]*_v_*, of equal magnitude and opposite sign. This means the AC of a chiral molecule can be defined by its specific optical rotation. Moreover, electronic circular dichroism (ECD) is another choice for the absolute configuration of a molecule which has chromospheres near the chiral center. Generally, the AC of a chiral molecule can be deduced directly from its ECD spectrum using semiempirical correlations. However, the relative method is based on some empirical chirality rules that sometimes involve exceptions which may lead to incorrect assignments. Fortunately, the increasing applicability of chiroptical methods in structural analysis has been remarkably facilitated by the development of methodology for *ab initio* predictions of chiroptical properties. With the aid of quantum-chemical calculations, one can not only confirm the known AC of compound with a single source of chirality by judging whether the predicted data match well with the corresponding measured data, but also identify the unknown AC when the calculated findings for a chosen configuration are in accordance with the experimental findings. Hence, experimental value combined quantum-chemical calculation has been used as an effective tool in the absolute configurational determination of chiral drug molecules, since they can help unambiguous to achieve in the correct assignment of AC.

Our group has focused on investigating and developing potent, highly selective and less toxic chiral drugs and meanwhile studying chiral molecular structure and the effect of stereochemistry on bioactivity. The main objective of our work is further study of the role of stereostructural pyridine-3-sulfonamide derivatives in bioactivity. The enantiomers of 5-bromo-2-chloro-*N*-(1-phenylethyl)pyridine-3-sulfonamide (**10a** and **10b**) have been synthesized and chosen as research candidates. In addition, in order to confirm the stereostructures and obtain deeper insights into the structural characteristics of chiral sulfonamides, XDR and the combination of measured and simulated optical properties (OR and ECD) have been performed to determine the ACs. Moreover, conformational analyses of the two compounds were conducted, and the structures optimized by a density functional theory (DFT) method were compared with the X-ray structures. Finally, effect of stereochemistry on the PI3Kα kinase and anticancer activity were evaluated.

## 2. Results and Discussion

### 2.1. Preparation of the Target Compounds

**Scheme 1:**
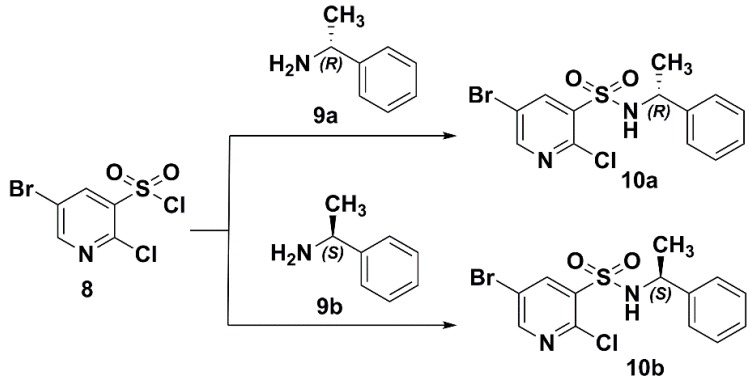
Synthetic procedures of **10a** and **10b**.

The target compounds were synthesized from 5-bromo-2-chloropyridine-3-sulfonyl chloride (**8**) and (*R*)-1-phenylethan-1-amine or (*S*)-1-phenylethan-1-amine (Scheme 1). Compound **8** was synthesized using the method in the literature [3].

### 2.2. X-ray Structure Analysis and Conformational Analyses

**Figure 3:**
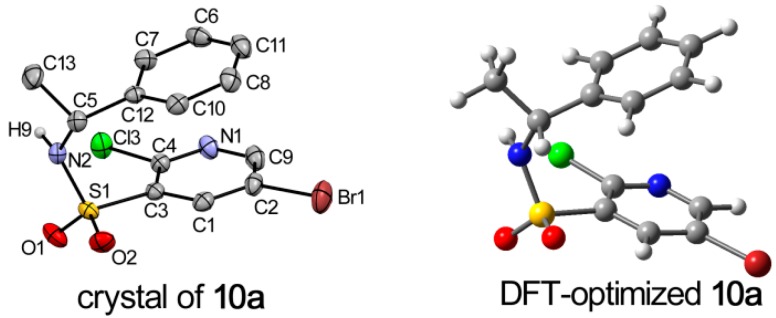
DFT-optimized and crystal structure of **10a**; displacement ellipsoids are drawn at the 30% probability level.

**Figure 4:**
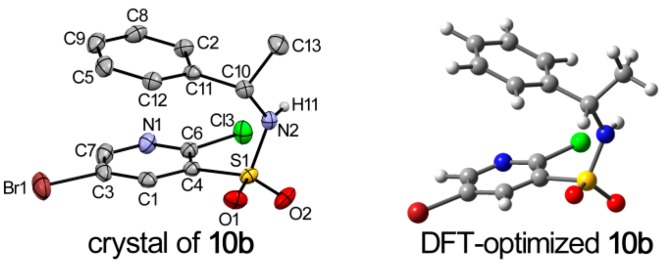
DFT-optimized and crystal structure of **10b**; displacement ellipsoids are drawn at the 30% probability level.

The crystals of **10a** and **10b** were grown by slow evaporation of chloroform under ambient conditions, and suitable crystals were obtained for crystallographic analysis. The measured values reveals that **10a** possesses a monoclinic crystal system with a P2_1_ space group (unit cell: *a* = 9.0403(6) Å, *b* = 7.4080(6) Å, *c* = 11.6060(9) Å). Compound **10b** also crystallizes in the monoclinic crystal system with the P2_1_ space group (unit cell: *a* = 9.063(3) Å, *b* = 7.423(3) Å, *c* = 11.614(4) Å). ORTEP diagrams of the molecular structures and the atomic numbering schemes of **10a** and **10b** are shown in Figure 3 and Figure 4, respectively. The hydrogen atoms were omitted for clarity. The crystallographic and refinement data are shown in Supplementary Material Table S1. As depicted by the figures in Figure 3 and Figure 4 the absolute configuration at the stereogenic center in the molecular structure **10a** is indeed *R* and in **10b** is *S*.

**Figure 5:**
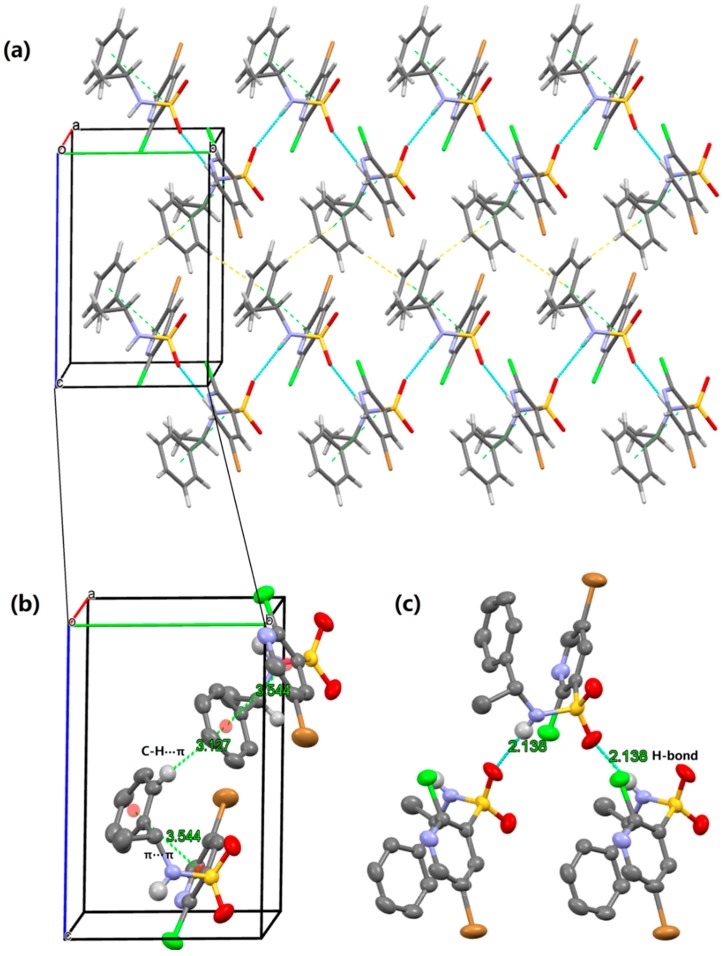
Crystal packing showing intermolecular N–H···O hydrogen bond, C—H···π and π-π interactions of **10a** as dashed lines.

To obtain deeper insight into the structure characteristics of the designed analogs, an X-ray structure analysis for **10a** and **10b** was performed. For compound **10a**, each molecule interacted with the other two molecules, and formed a trimer via N(2)–H(9)···O(1)i (i = 1 − x, −1/2 + y, 2 − z) and O(1)···H(9)–N(2)ii (ii = 1 − x, 1/2 + y, 2 − z) hydrogen bonds (Table 1). Moreover, in the structure can be found a C—H···π interactions expanding in chains of supramolecular layer propagating along crystallographic plane (Figure 5). In addition to the afore-described interactions, a weak π-π packing existed between substituted pyridine and benzene plane in a molecule. The perpendicular distance was found with distances of Cg on pyridine ring and Cg’ on benzene ring being 3.544 Å. The above-mentioned interactions played important role in view of the stability of the crystal structure. In the crystal of **10b**, the contacts and packing are demonstrated in the similar manner, as shown in Supplementary Material Figure S1 and Table S2.

**Figure 6:**
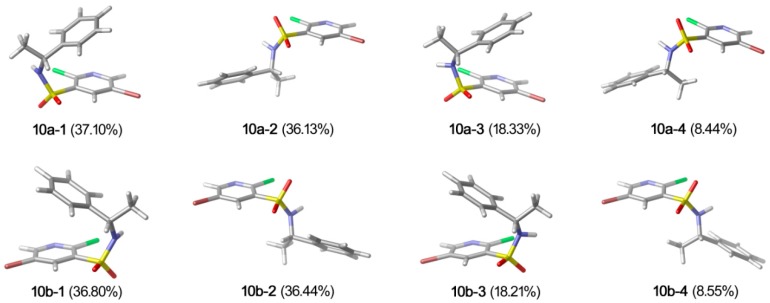
Relatively stable conformers of **10a** and **10b**.

The conformation of a molecule critically influences its physical and chemical properties [14,15,16]. Thus, reliable conformational analysis plays a key role in the understanding of structure. Initial conformational searching of compound **10a** and **10b** were performed by Spartan 08 program [17] with MMFF [18,19] molecular mechanics force field. Then, geometry optimizations and frequency calculation of all the possible conformers were performed by using DFT/B3LYP/6-311++G** [20,21] in Gaussian 09 package [22]. From the relative free energies, the percentage population of each conformation in a room-temperature equilibrium mixture can be predicted. The relative Gibbs free energies and Boltzmann distribution of **10a** and **10b** are shown in Table 2, and the conformers of **10a** and **10b** are presented in Figure 6.

**Table 1:** Hydrogen-bond geometry (Å, °) of **10a**.

D—H···A	D—H	H···A	D···A	D—H···A
N2—H9···O1 ^i^	0.871	2.138	3.004	172.29
O1···H9—N2 ^ii^	0.871	2.138	3.004	172.29

Symmetry code: (i) 1 − x, −1/2 + y, 2 − z; (ii) 1 − x, 1/2 + y, 2 − z.

**Table 2:** Gibbs free energies (G), relative Gibbs free energies (△G) ^a^ and Boltzmann weighting factor (*P*%) ^b^ of **10a** and **10b** conformers by using the DFT/B3LYP/6-311++G(2d, p) method.

Conformer	G(kcal·mol^−1^)	△G(kcal·mol^−1^)	*P_i_*%
**10a-1**	−2632474.97	0.0000	37.10
**10a-2**	−2632474.96	0.0157	36.13
**10a-3**	−2632474.56	0.4135	18.33
**10a-4**	−2632474.11	0.8678	8.44
**10b-1**	−2632474.97	0.0000	36.80
**10b-2**	−2632474.96	0.0056	36.44
**10b-3**	−2632474.55	0.4123	18.21
**10b-4**	−2632474.11	0.8553	8.55

^a^ which related to the most stable conformer; ^b^ Boltzmann weighting factor (*P_i_*%) based on ∆G.

In the case of **10a**, conformer **10a-1** (37.10%), **10a-2** (36.13%), **10a-3** (18.33%) and **10a-4** (8.44%) are significantly populated at room temperature. The ground-state energies of **10a-1** and **10a-2** are similar, in addition, **10a-1** and **10a-3** only a slight difference between the orientation of hydrogen atom in sulfonamide group is observed. Similar results were found for the enantiomer **10b**, and the major contributions were from conformer **10b-1** (36.80%), **10b-2** (36.44%), **10b-3** (18.21%) and **10b-4** (8.55%). The crystal structures of **10a** and **10b** were compared with the DFT-optimized structures. Among all the conformers of **10a** and **10b**, conformer **10a-1** and **10b-1** were in accord with the crystalline structure, respectively. Some selected experimental and calculated geometry parameters for **10a** and **10b** are listed in Table 3 (all parameters are shown in Supplementary Material Table S3). As expected, most of the calculated geometry parameters for the two compounds are close to the X-ray data.

**Table 3:** Selected experimental and calculated geometry parameters for **10a** and **10b**.

Bond Angle [°]	Exp. (10a)	Calcd. ^a^	Difference
C(12)C(5)C(13)	111.1	112.2	1.1
C(12)C(5)N(2)	113.6	114.9	1.3
C(13)C(5)N(2)	107.6	107.7	0.1
C(5)N(2)S(1)	121.1	123.4	2.3
C(5)N(2)H(9)	113	117.8	4.8
H(9)N(2)S(1)	120	112.3	−7.7
Bond angle [°]	Exp. (**10b**)	Calcd. ^b^	Difference
C(13)C(10)C(11)	111.0	112.2	1.2
N(2)C(10)C(11)	113.3	114.9	1.6
C(13)C(10)N(2)	107.7	107.7	0
S(1)N(2)C(10)	121.2	123.4	2.2
H(11)N(2)C(10)	119	117.8	−1.2
H(11)N(2)S(1)	114	112.3	−1.7

^a^ Calculated geometry parameters for conformer **10a-1**; ^b^ Calculated geometry parameters for conformer **10b-1**.

### 2.3. ECD Analysis

**Figure 7:**
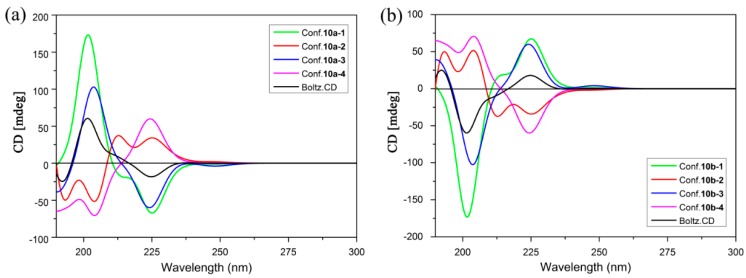
Calculated ECD spectra of individual conformers of (**a**) **10a** and (**b**) **10b**.

The ECD spectra of the stable conformers of **10a** and **10b** in methanol were calculated by using the PCM with time-dependent density functional theory (TD-DFT) method at the CAM-B3LYP/aug-cc-PVDZ level. All of the simulated spectra of the lowest-energy conformations were averaged according to the Boltzmann distribution theory by the Specdis program (Figure 7) [23].

**Figure 8:**
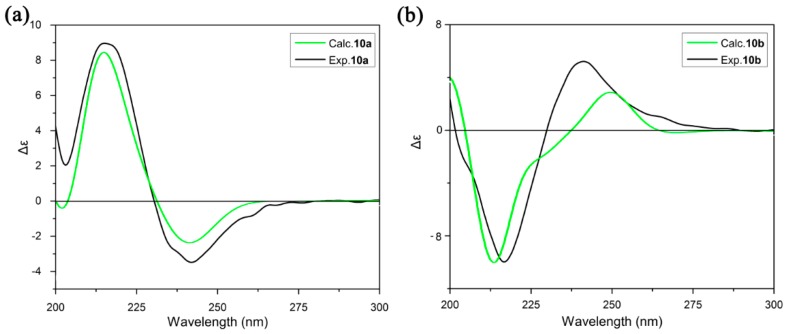
Experimental and calculated (TD-DFT/CAM-B3LYP/aug-cc-PVDZ) ECD spectra of (**a**) **10a** and (**b**) **10b**.

In the experimental ECD spectrum of **10a**, a strong positive cotton effect (CE) is observed at 215 nm, a negative CE at 242 nm. In the case of **10b**, the experimental ECD spectrum shows a positive CE at 242 nm and a strong negative CE at 215 nm. The simulated ECD spectrum for **10a** and **10b** in MeOH which have been re-plotted with population weighting along with experimental spectrum are shown in Figure 8. It can be seen that TD-DFT calculations provided excellent agreement to the measured ECD band shape. The calculated ECD of **10a** showed a strong positive CE at 215 nm and a negative CE at 242 nm, similar to the curve in the experimental spectrum of **10a**. Meanwhile, the CEs at 215 and 242 nm in the experimental ECD spectrum of **10b** were satisfactorily reproduced by the simulated ECD spectrum of **10b**. Notwithstanding some small discrepancies, this result supports the assignment of an R configuration to **10a** and an S configuration to **10b**.

The molecular conformations and the stereochemistry affect the CEs greatly. This can be seen from the calculated ECDs of individual conformers of **10a** and **10b** in Figure 7. The calculated ECD of **10a-1** shows the same signs of CEs at 215 and 242 nm as that of **10a-2** with only slightly weaker magnitude at 215 nm. While the simulated ECD curve of **10a-3** and **10a-4** presents opposite sign of CEs at 215 and 242 nm. For compound **10b**, the relationship between individual conformation and CEs would be explicated by the similar way. **10b-1** and **10b-2** show the almost identical ECD spectra to Boltz.CD, while **10b-3** and **10b-4** exhibit the converse curves. Taking above-mentioned analyses into account, the ECDs of individual conformers are based on the comprehensive factors of conformation and configuration.

**Figure 9:**
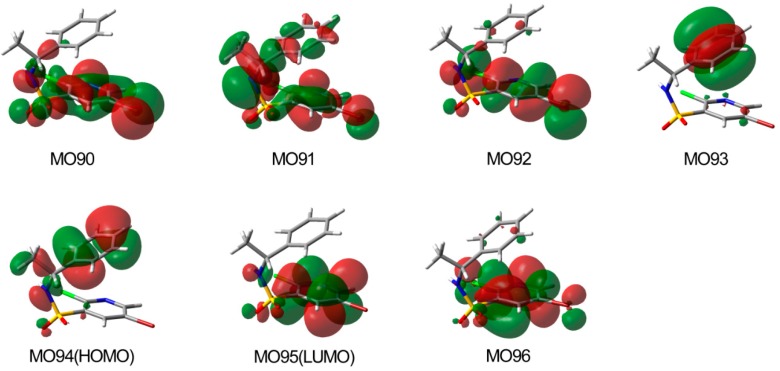
Molecular orbitals involved in the electronic transitions of **10a**.

The origin of the CEs in ECD spectra of **10a** and **10b** could be explained by molecular orbital (MO) analysis at the same level as the ECD calculation. Figure 9 shows the MOs of **10a** mainly involved in the electronic transitions used to assign the experimental bands. The positive CE at 215 nm has contributions from the electronic transition from MO90 and MO91 to LUMO95 (LUMO = lowest unoccupied MO) and from MO93→MO96. In addition, the negative CE at 242 nm in the experimental spectrum might be caused by the transition from MO92 to MO96 and LUMO95 with a contribution from a HOMO94→LUMO95 (π→π*; HOMO = highest occupied MO) excitation. In the case of **10b** (Figure S2), the CEs at 215 and 242 nm in ECD spectrum are dominated by the same transitions as the CEs at 215 and 232 nm for **10a**.

### 2.4. Optical Rotation Analysis

Specific optical rotations of target compounds at 589.3 nm have been predicted using the B3LYP method and 6-311++G** as basis set, with the measured data given in Table 4. Obviously, theoretical [*α*] findings show good agreement with experimental optical rotation under this circumstance. It is worth noting that experimental and calculated data confirm that the ACs of target compounds are R(+)/S(−).

**Table 4:** The calculated and experimental optical rotation (OR) of **10a** and **10b**.

No.	Theoretical OR/°	Experimental OR/°
**10a**	73.0	58.2
**10b**	−89.9	−65.9

### 2.5. Binding Model Analysis

**Figure 10:**
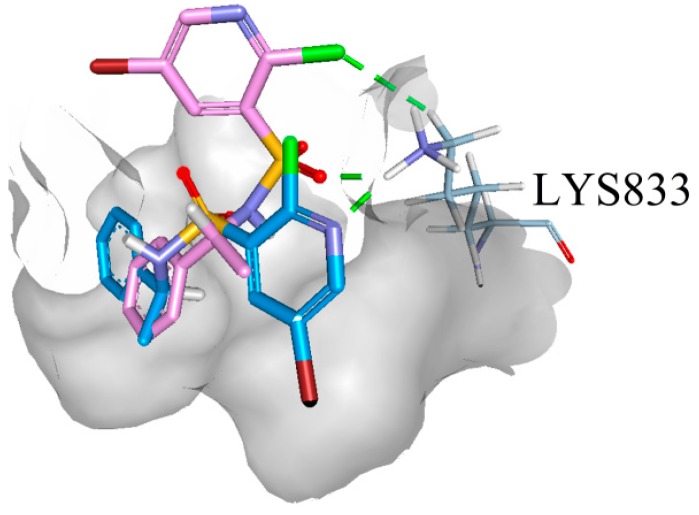
The PI3Kγ active site in complex with compound **10a** (pink) and **10b** (blue).

To further elucidate the binding mode of compounds, a detailed docking analysis was performed. As previously mentioned, the pyridine-3-sulfonamide fragment exhibits a remarkable effect in the PI3K inhibitor. Therefore, the PI3Kγ crystal structure (PDB ID code: 3L08) was selected as the docking model [7]. The docking simulation was conducted using Glide XP (Schrödinger 2014), since Glide uses a hierarchical series of filters to search for possible locations of the ligand in the active-site region of the receptor. The shape and properties of the receptor are represented on a grid by several different sets of fields that provide progressively more accurate scoring of the ligand poses. The image files were generated using the Accelrys DS visualizer 4.0 system. The binding model was exemplified by the interaction of compound **10a** and **10b** with PI3Kγ. As shown in Figure 10, the chlorine atom and the oxygen atom of the sulfonamide in **10a** formed two hydrogen-bonding interactions with LYS833. A hydrogen bond between the nitrogen atom of pyridine ring and LYS833 was detected for compound **10b**. Obviously, different binding moieties were observed between **10a** and **10b**, which was originated from the stereogenic center, so different biological activities would be detected.

### 2.6. PI3Kα Kinase Assay

Compounds **10a** and **10b** were evaluated for their PI3Kα enzymatic activities using a homogeneous time-resolved fluorescence (HTRF) assay. GDC-0941 (IC_50_ = 3.74 ± 0.26 nM) was used as a positive control in this assay, compounds **10a** and **10b** inhibited PI3Kα kinase with IC_50_ values of 1.08 ± 0.09 and 2.69 ± 0.22 μM, respectively. This is consistent with docking analysis.

### 2.7. In Vitro Cytotoxicity

The cytotoxicity of the target compounds were evaluated against cancer cells line HepG2 (human hepatocellular carcinoma) by using MTT assay. GDC-0941 (IC_50_ = 1.23 ± 0.17 μM) was used as the positive control. In the *in vitro* antitumor activity studies, both **10a** and **10b** were found to be biologically active; the inhibitory activity of **10a** was a little better than that of **10b**, and the IC_50_ values were 9.26 ± 0.89 and 24.17 ± 2.34 μM, respectively.

## 3. Experimental Section 

### 3.1. General Remarks

Melting points were obtained on a Büchi Melting Point B-540 apparatus (Büchi Labortechnik, Flawil, Switzerland) and were uncorrected. The IR spectrum of the title compound was recorded in the region 4000–400 cm^−1^ using KBr pellet technique with 1.0 cm^−1^ resolution on a Bruker IFS-55V IR spectrometer (Bruker, Ettlingen, Germany). Mass spectra (MS) were taken in ESI mode on Agilent 1100 LC-MS (Agilent Technologies, Palo Alto, CA, USA). ^1^H-NMR and ^13^C-NMR spectra were recorded on Bruker AVANCE-600 MHz NMR spectrometer (Bruker) with tetramethylsilane (TMS) as an internal standard. The optical rotations were measured in CHCl_3_ using a Rudolph Autopol IV automatic polarimeter (Rudolph Research Analytical, Hackettstown, NJ, USA). ECD spectra were recorded on a MOS-450 circular dichroism spectrometer spectropolarimeter (Biologic, France). The spectra were measured at solute concentration of 0.2 mg·mL^−1^ using a 1 mm path length quartz cuvette at 25 °C in a wavelength range of 190 to 400 nm, and methanol was used as solvent. The X-ray diffraction data of the crystals of **10a** and **10b** were recorded with a Bruker P4 X-diffractometer; the data were collected by using graphite-monochromated Mo-Kα radiation (λ = 0.71073 Å) at 293 K. For **10a** and **10b**, data collection: APEX2 [24]; cell refinement: SAINT [25]; program used to solve structure: SHELXS-97 [26]; program used to refine structure and draw molecular figures: SHELXTL-97 [26]; program used to measure centroid-centroid distance: Mercury 2.3 [27]. All materials were obtained from commercial suppliers and were used without further purification.

### 3.2. Materials

Pyridine and dichloromethane were purchased from the Aldrich, Co. Ltd. and used after dehydration with 4 Å molecular sieves. (*R*)-1-phenylethan-1-amine, (*S*)-1-phenylethan-1-amine and 5-bromo-2-chloropyridin-3-amine were purchased from the Tokyo Chemical Industry Co. Ltd. All reagents were of analytical grade and are commercially available. For TLC analysis, precoated plates of silica gel 60 F254 were used, and spots were visualized with UV light.

CCDC-1406603 (for **10a**) and -1406604 (for **10b**) contain the supplementary crystallographic data for this paper. These data can be obtained free of charge via http://www.ccdc.cam.ac.uk/conts/ retrieving.html (or from the CCDC, 12 Union Road, Cambridge CB2 1EZ, UK; Fax: +44 1223 336033; E-mail: deposit@ccdc.cam.ac.uk).

### 3.3. Synthesis of (R)-5-Bromo-2-chloro-N-(1-phenylethyl)pyridine-3-sulfonamide *(**10a**)*

A mixture of 5-bromo-2-chloropyridine-3-sulfonyl chloride (5.00 g, 17.19 mmol; prepared according to the literature method) [3], (*R*)-1-phenylethan-1-amine (2.08 g, 17.19 mmol), and pyridine (4.08 g, 51.57 mmol) in dichloromethane (50 mL) was stirred at room temperature for 3 h. The reaction mixture was poured into 1 N hydrochloric acid (60 mL), and then extracted with dichloromethane. The organic phase was then dehydrated with anhydrous sodium sulfate and concentrated at reduced pressure, and the residue was crystallized from methanol to afford a light yellow powder in a yield of 78% (5.04 g). m.p.: 173.4–174.9 °C. [*α*]_D_ = 58.2 (*c* = 1, CHCl_3_). IR (KBr pellets): *ν* 3284.3 (NH), 3110.3, 3063.7, 3043.4, 3024.4 (ArH), 2980.5, 2933.4 (CH_3_), 1632.3 (C=N), 1601.0 (NH), 1585.3, 1540.2, 1493.5 (C=C), 1424.7 (CH_3_ asym), 1356.3 (CH_3_ sym), 1340.2 (SO_2_ asym), 1166.6 (SO_2_ sym), 1120.6 (C-N), 1109.6 (C-Br), 869.3, 851.7, 816.8, 728.3 (ArH), 768.2 (C-Cl), 703.8 (C-Br) cm^−1^. ^1^H-NMR (600 MHz, CDCl_3_) δ 8.36 (d, *J* = 2.4 Hz, 1H), 7.96 (d, *J* = 2.4 Hz, 1H), 7.10 (t, *J* = 5.5 Hz, 3H), 7.08–7.04 (m, 2H), 5.55 (d, *J* = 7.3 Hz, 1H), 4.58 (p, *J* = 7.0 Hz, 1H), 1.55 (d, *J* = 7.0 Hz, 3H). ^13^C NMR (150 MHz, CDCl_3_) δ 152.54 (C-9), 145.68 (C-4), 141.51 (C-12), 139.66 (C-1), 136.35 (C-3), 128.50 (C-6,8), 128.26 (C-7,10), 126.26 (C-11), 118.99 (C-2), 54.85 (C-5), 22.7 (C-13). MS (ESI+): *m/z* = 376.9 [M + H]^+^.

### 3.4. Synthesis of (S)-5-Bromo-2-chloro-N-(1-phenylethyl)pyridine-3-sulfonamide *(**10b**)*

The similar procedure was conducted to afford **10b** by (S)-1-phenylethan-1-amine. m.p.: 174.5–175.5 °C. [*α*]_D_ = −65.9 (*c* = 1, CHCl_3_). IR (KBr pellets): *ν* 3284.3 (NH), 3110.2, 3063.6, 3043.4, 3024.4 (ArH), 2980.5, 2933.6 (-CH_3_), 1631.9 (C=N), 1600.9 (NH), 1585.2, 1540.1, 1493.5 (C=C), 1424.7 (CH_3_ asym), 1356.2 (CH_3_ sym), 1340.1 (SO_2_ asym), 1167.3 (SO_2_ sym), 1120.6 (C-N), 1109.5 (C-Br), 869.3, 851.7, 816.8, 728.2 (ArH), 768.1 (C-Cl), 703.7 (C-Br) cm^−1^. ^1^H-NMR (600 MHz, CDCl_3_) δ 8.36 (d, *J* = 2.4 Hz, 1H), 7.96 (d, *J* = 2.4 Hz, 1H), 7.10 (t, *J* = 5.4 Hz, 3H), 7.08–7.03 (m, 2H), 5.51 (d, *J* = 7.3 Hz, 1H), 4.58 (p, *J* = 7.1 Hz, 1H), 1.55 (d, *J* = 7.0 Hz, 3H). ^13^C-NMR (150 MHz, CDCl_3_) δ 152.54 (C-7), 145.67 (C-6), 141.51 (C-11), 139.65 (C-1), 136.35 (C-4), 128.50 (C-5,8), 128.27 (C-2,12), 126.26 (C-9), 119.00 (C-3), 54.86 (C-10), 22.7 (C-13). MS (ESI+): *m/z* = 376.9 [M + H]^+^.

### 3.5. Computational Details

The conformational analysis was firstly performed by arbitrarily fixing the absolute configuration of the target compound, using the Spartan 08 program [17] with MMFF [18,19] (molecular mechanics force field). Then all of the possible conformers were optimized at B3LYP level of theory using 6-311++G** [20,21] basis sets under PCM model [28,29] in Gaussian 09 package [22]. Frequency calculations based on previously optimized geometries were performed in order to ensure the minimum energy of the structure. Relative population of each conformer was valued on the basis of Boltzmann weighting factor at 298 K which was also calculated at the same level in order to simulate OR and ECD.

### 3.6. PI3Kα Kinase Assay

The PI3Kα kinase activities were evaluated using homogeneous time-resolved fluorescence (HTRF) assays as previously reported protocol [30,31]. Briefly, 20 μg/mL poly (Glu, Tyr) 4:1 (Sigma-Aldrich, St. Louis, MO, USA) was preloaded as a substrate in 384-well plates. Then 50 μL of 10 mM ATP (Invitrogen, Waltham, MA, USA) solution diluted in kinase reaction buffer (50 mM HEPES, pH 7.0, 1 mM DTT, 1 mM MgCl_2_, 1 mM MnCl_2_, and 0.1% NaN_3_) was added to each well. Various concentrations of compounds were diluted in 10 μL of 1% DMSO (*v*/*v*), with blank DMSO solution as the negative control. The kinase reaction was initiated by the addition of purified tyrosine kinase proteins diluted in 39 μL of kinase reaction buffer solution. The incubation time for the reactions was 30 min at 25 °C and the reactions were stopped by the addition of 5 μL of Streptavidin-XL665 and 5 μL Tk Antibody Cryptate working solution to all of wells. The plate was read using Envision (Perkin Elmer, Waltham, MA, USA) at 320 nm and 615 nm. IC_50_ values were calculated from the inhibition curves.

### 3.7. MTT Assay in Vitro

The anti-proliferative activities of compounds **10a** and **10b** were evaluated against human HepG2 cell line using the standard MTT assay *in vitro*. The cancer cell line was cultured in minimum essential medium (MEM) by supplement with 10 % fetal bovine serum (FBS). Approximate 4 × 10^3^ cells, suspended in MEM medium, were plated onto each well of a 96-well plate and incubated in 5% CO_2_ at 37 °C for 24 h. The tested compounds at the indicated final concentrations were added to the culture medium and the cell cultures were continued for 72 h. Fresh MTT was added to each well at a terminal concentration of 5 mg/mL, and incubated with cells at 37 °C for 4 h. The formazan crystals were dissolved in 100 mL DMSO for each well, and the absorbancy at 492 nm (for absorbance of MTT formazan) and 630 nm (for the reference wavelength) were measured with an ELISA reader. All compounds were tested three times in each of the cell lines. The results expressed as IC_50_ (inhibitory concentration 50%) were the averages of three determinations and calculated by using the Bacus Laboratories Incorporated Slide Scanner (Bacus Laboratories Inc., Lombard, IL, USA) software.

## 4. Conclusions

*R*- and *S*-isomers of 5-bromo-2-chloro-*N*-(1-phenylethyl)pyridine-3-sulfonamide have been synthesized. The ACs of the isomers were determined by X-ray diffraction, and the assignments of the ACs were further validated by comparing experimental and theoretical ECD spectra and OR. In addition, tests of the PI3Kα kinase and *in vitro* anticancer activities of the compounds have verified through docking analysis that the activity of the *R* isomer is better than that of *S* stereoisomer.

## Supplementary Materials

The supplementary crystallographic data for **10a** and **10b** reported in this paper have been deposited with the Cambridge Crystallography Data Centre, 12 Union Road, Cambridge CB22 1EZ, UK (Fax: +44 1223 336 033; E-mail: deposit@ccdc.ca-m.ac.uk or http://www.ccdc.cam.ac.uk) and are available freely on request quoting the deposition number CCDC-1406603, CCDC-1406604 for **10a** and **10b**, respectively. IR, MS, ^1^H and ^13^C-NMR spectra of **10a** and **10b**, the detailed crystal geometry parameters for **10a** and **10b** are listed. Supplementary materials can be accessed at: http://www.mdpi.com/1420-3049/20/11/19740/s1.
