# Supplementary material for: Synthesis, Crystal Structure, Absolute Configuration and Antitumor Activity of the Enantiomers of 5-Bromo-2-chloro-N-(1-phenylethyl)pyridine-3-sulfonamide
Source: Molecules. 2015 Nov 24;20(11):20926–38. doi: 10.3390/molecules201119740 (PMC6332033; doi:10.3390/molecules201119740)
Supplement: Supplementary file 1 [file molecules-20-19740-s001.pdf]

Supplementary Materials: Synthesis, Crystal Structure, Absolute Configuration and Antitumor Activity of the Enantiomers of 5-Bromo-2-chloro-*N*-(1-phenylethyl)pyridine-3-sulfonamide

Zhixu Zhou, Linwei Li, Ning Yan, Lei Du, Changshan Sun and Tiemin Sun

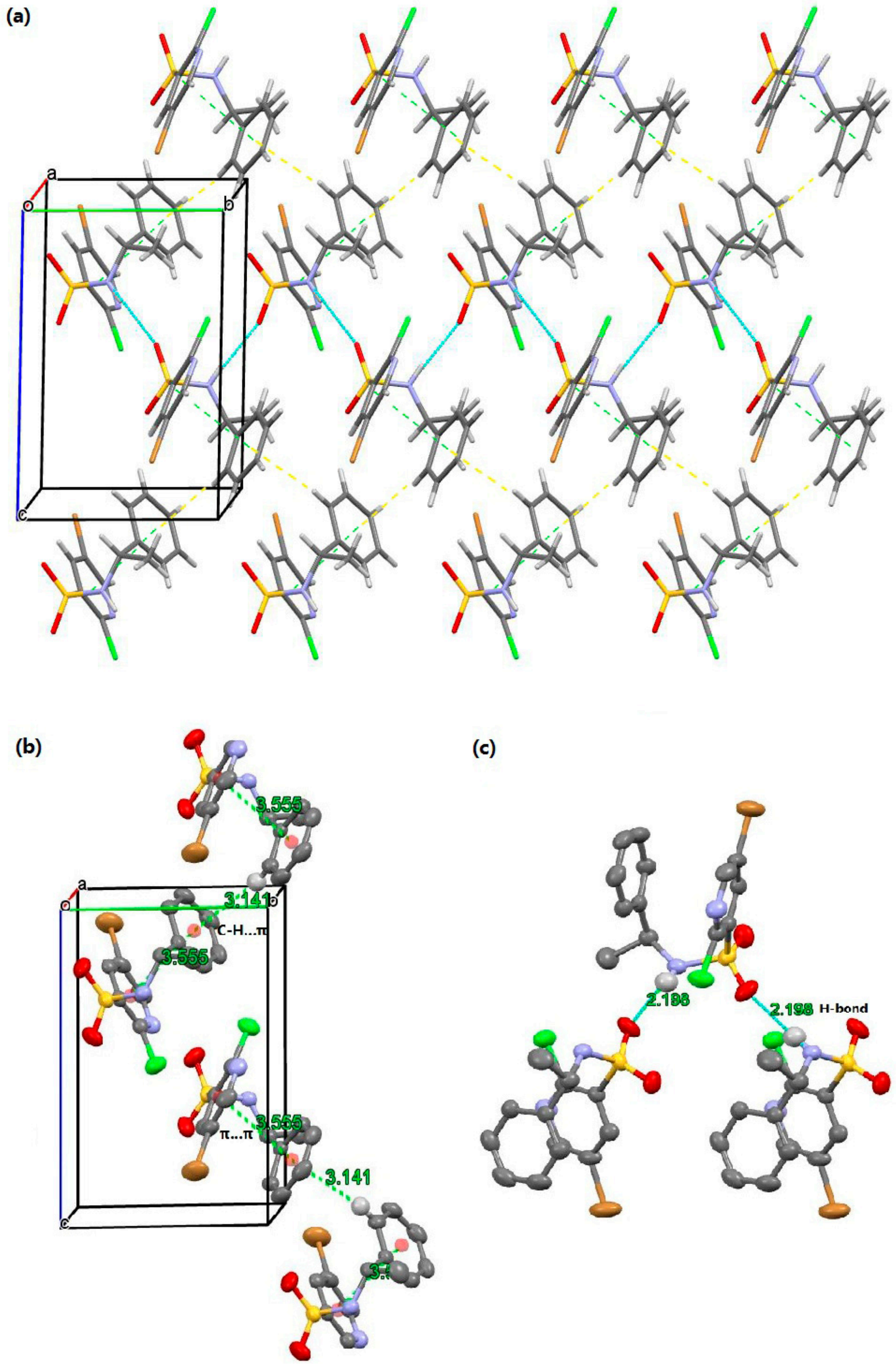

Figure S1. Crystal packing showing intermolecular N–H···O hydrogen bond, C—H···π and π-π interactions of **10b** as dashed lines.

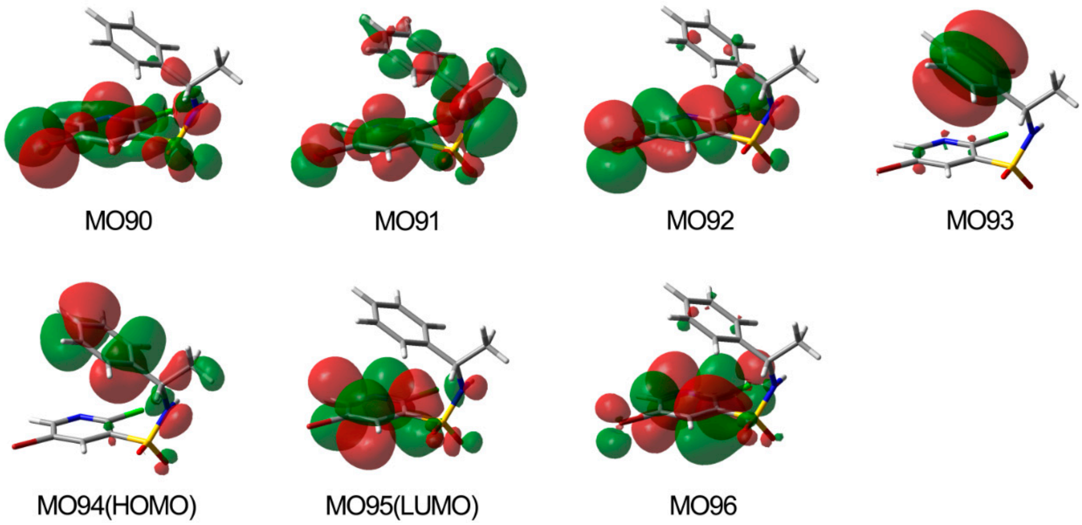

Figure S2. Molecular orbitals involved in the electronic transitions of **10b**.

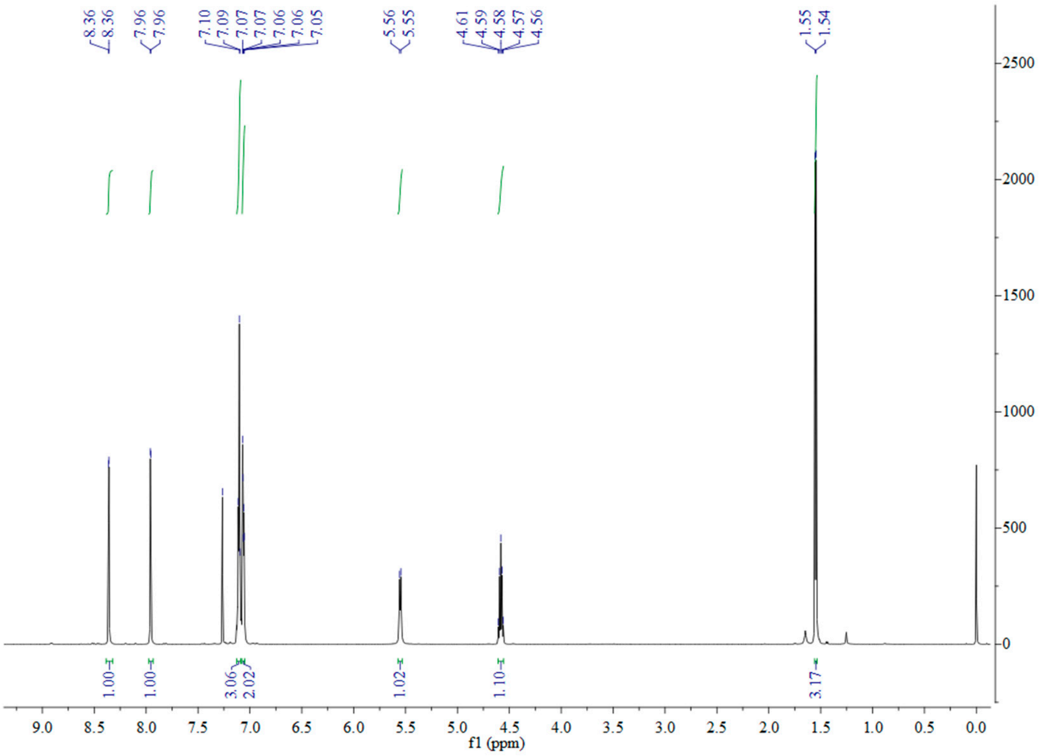

Figure S3. ^{1}H-NMR of **10a**.

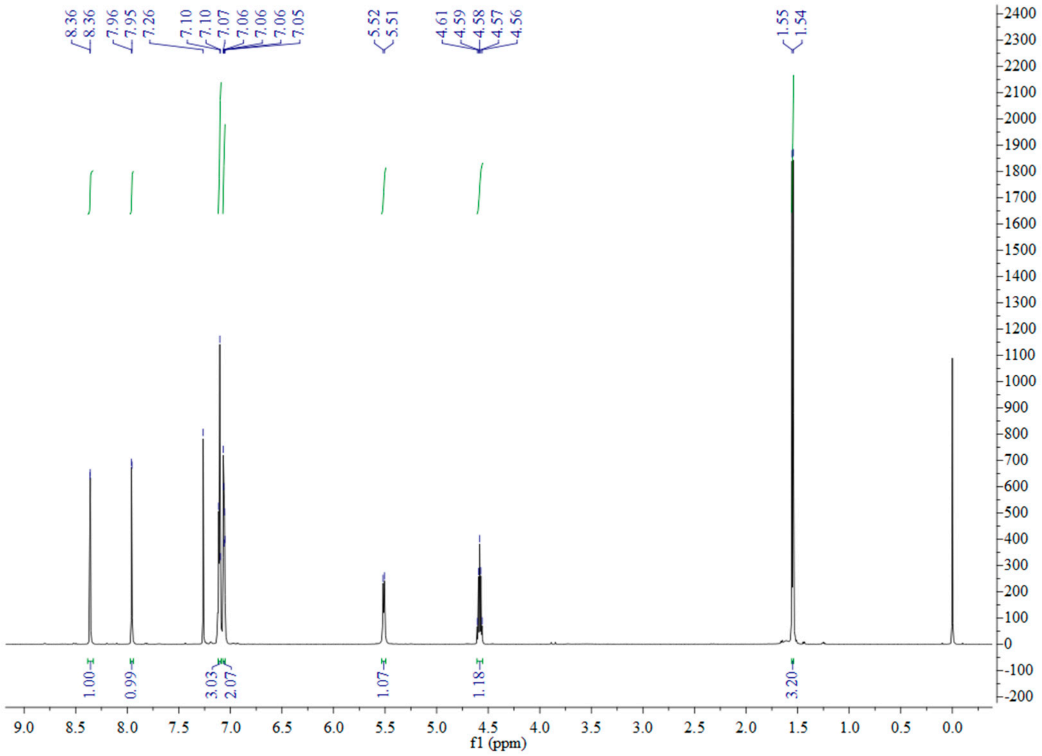

Figure S4. ^{1}HNMR of **10b**.

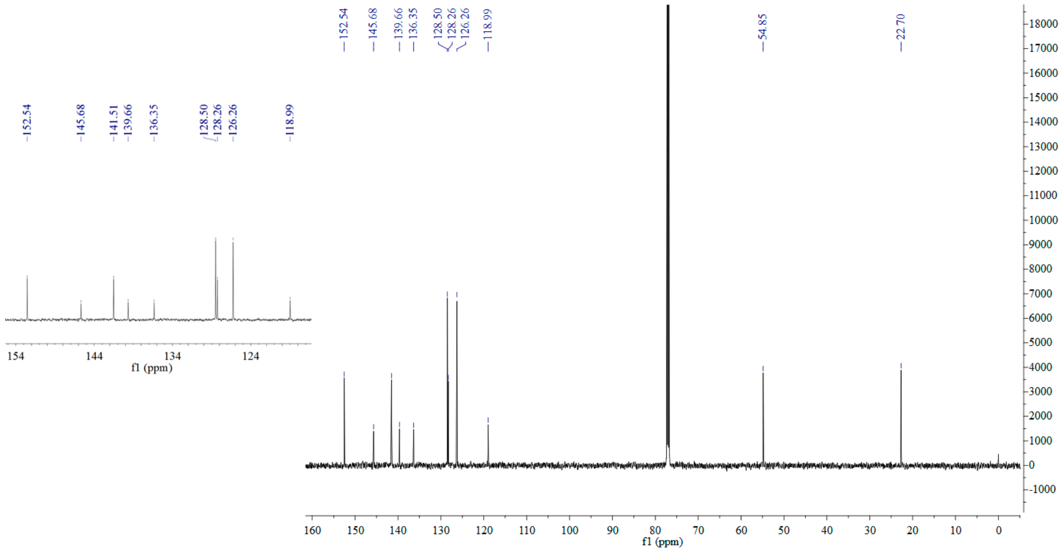

Figure S5. ^{13}C-NMR of **10a**.

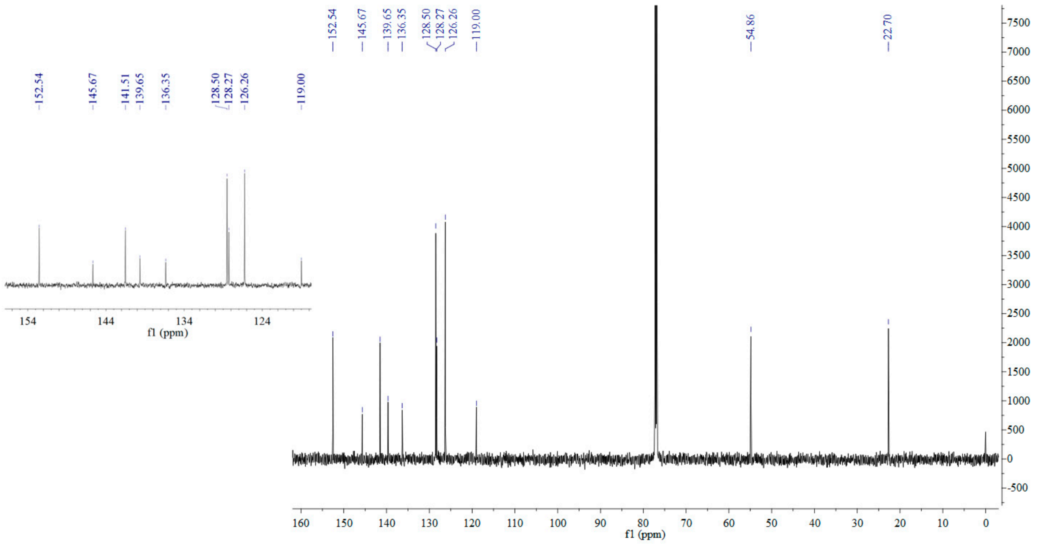

Figure S6. ^{13}C-NMR of **10b**.

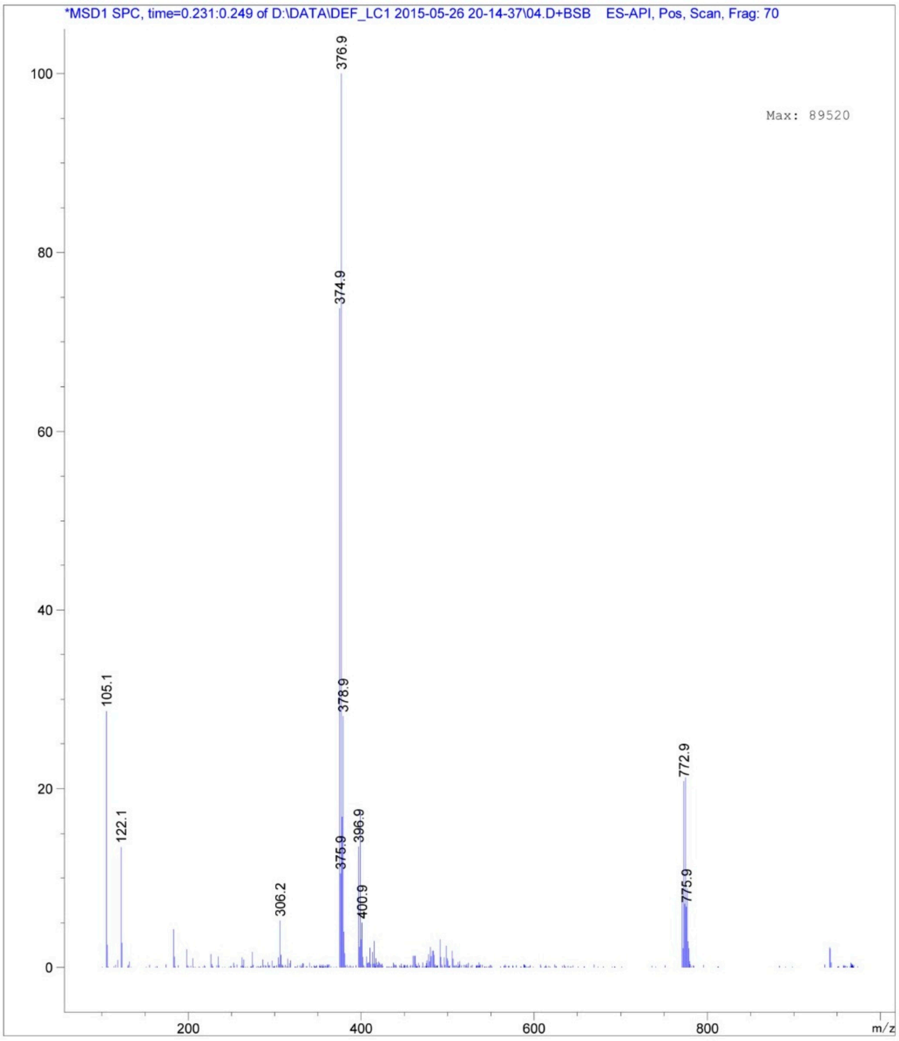

Figure S7. MS of **10a**.

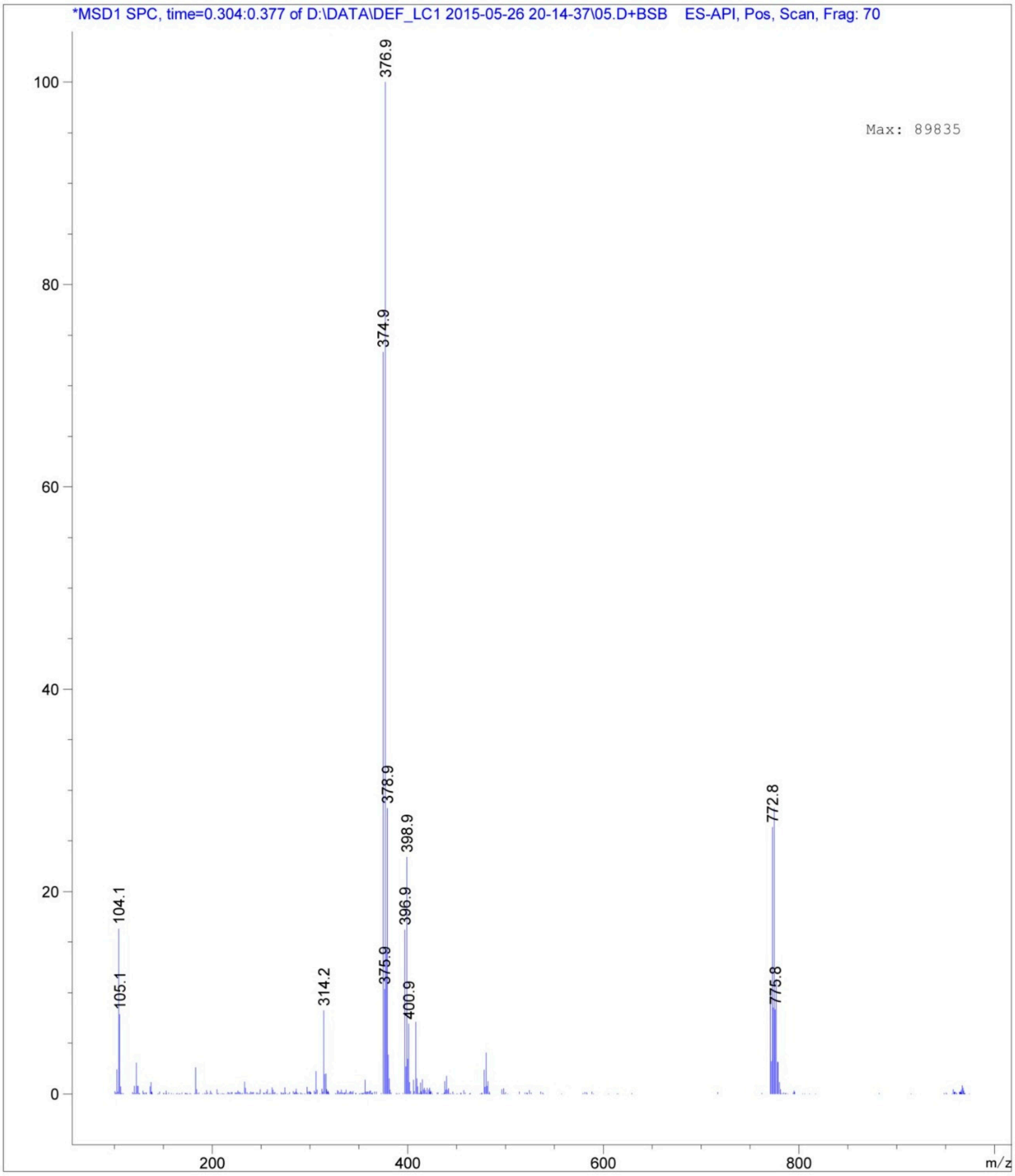

Figure S8. MS of **10b**.

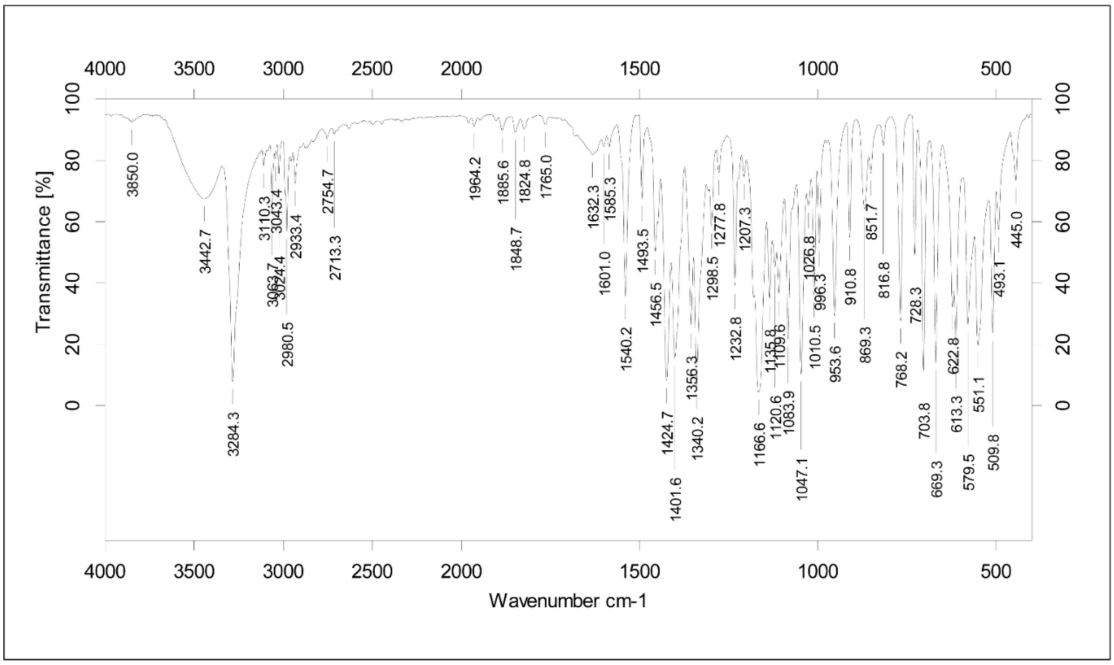

Figure S9. IR of **10a**.

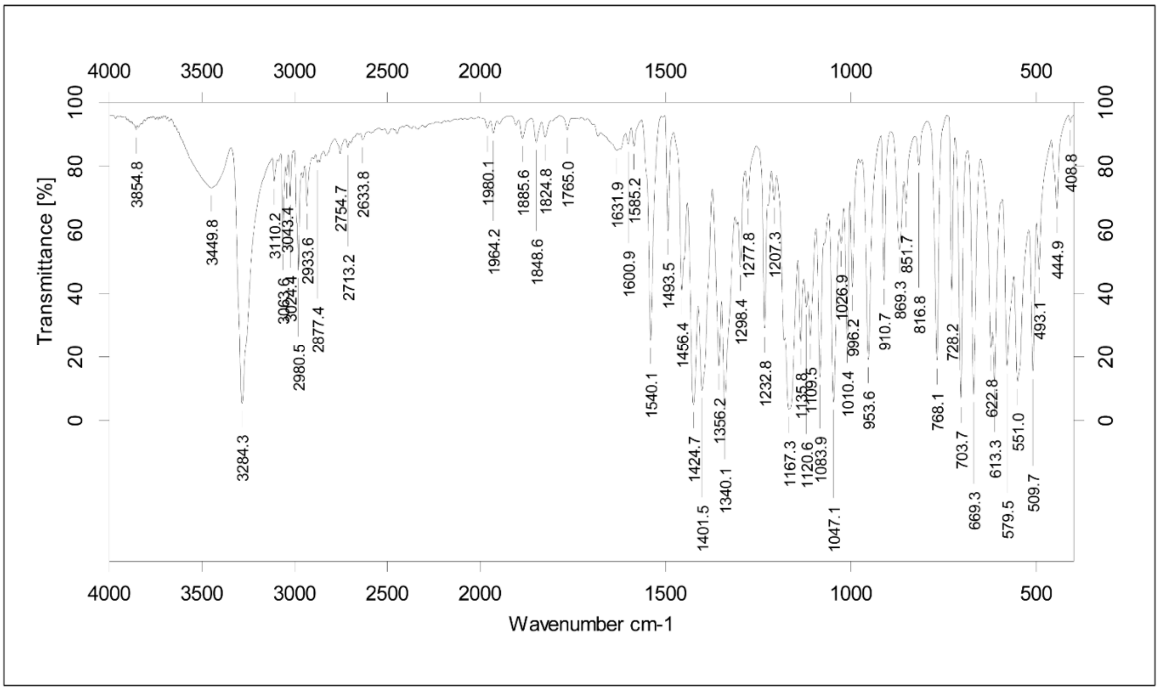

Figure S10. IR of **10b**.

Table S1. Crystal data and refinement details for **10a** and **10b**.

Derivatives	10a	10b
Empirical formula	$C_{13}H_{12}BrClN_2O_2S$	$C_{13}H_{12}BrClN_2O_2S$
Formula weight	375.67	375.67
CCDC number	1406603	1406604
Temperature (K)	296	296
Crystal color	colourless	colourless
Crystal system	monoclinic	monoclinic
Space group	P 2_1	P 2_1
a (Å)	9.0403(6)	9.063(3)
b (Å)	7.4080(5)	7.423(3)
c (Å)	11.6060(9)	11.614(4)
α (º)	90.00	90.00
β (º)	108.897(2)	108.668(8)
γ (º)	90.00	90.00
V ($Å^{-3}$)	735.37(9)	740.1(5)
Z	2	2
S	0.963	0.997
R1, wR_2	0.0296, 0.0675	0.0310, 0.0746
Data completeness	1.74/0.94	1.76/0.95
Theta (max)	28.630	28.480

Table S2. Hydrogen-bond geometry (Å, °) of **10b**.

D—H…A	D—H	H···A	D···A	D—H···A
N2—H11…O2 ^{i}	0.822	2.198	3.006	167.87
O2…H11—N2 ^{ii}	0.822	2.198	3.006	167.87
Symmetry code: (i) 2 − x, 1/2 + y, 1 − z; (ii) 2 − x, −1/2 + y, 1 − z.				

Table S3. Selected experimental and calculated geometry parameters for **10a** and **10b**.

Bond Distances (Å)	Exp. (10a)	Cal. (10a) [a]	Dif.	Bond Distances (Å)	Exp. (10b)	Cal. (10b)[b]	Dif.
Br(1)-C(2)	1.886	1.906	0.02	H(11)-N(2)	0.82	1.013	0.19
C(1)-C(2)	1.375	1.386	0.01	C(13)-H(13A)	0.961	1.093	0.13
C(1)-C(3)	1.384	1.388	0.00	C(13)-H(13B)	0.960	1.091	0.13
C(1)-H(3)	0.88	1.08	0.20	C(13)-H(13C)	0.960	1.091	0.13
C(2)-C(9)	1.371	1.387	0.02	C(13)-C(10)	1.526	1.522	0.00
C(3)-C(4)	1.392	1.399	0.01	Br(1)-C(3)	1.885	1.906	0.02
C(3)-S(1)	1.790	1.812	0.02	S(1)-C(4)	1.798	1.812	0.01
C(4)-Cl(3)	1.731	1.755	0.02	S(1)-N(2)	1.618	1.643	0.03
C(4)-N(1)	1.310	1.314	0.00	S(1)-O(2)	1.431	1.446	0.02
C(5)-C(12)	1.514	1.522	0.01	S(1)-O(1)	1.429	1.441	0.01
C(5)-C(13)	1.523	1.532	0.01	Cl(3)-C(6)	1.730	1.755	0.03
C(5)-H(7)	1.01	1.089	0.08	C(1)-H(1)	0.930	1.081	0.15
C(5)-N(2)	1.482	1.477	−0.01	C(1)-C(3)	1.380	1.386	0.01
C(6)-C(7)	1.383	1.392	0.01	C(1)-C(4)	1.384	1.388	0.00
C(6)-C(11)	1.370	1.390	0.02	C(2)-H(2)	0.929	1.085	0.15
C(6)-H(11)	0.90	1.084	0.18	C(2)-C(8)	1.389	1.390	0.00
C(7)-C(12)	1.401	1.394	-0.01	C(2)-C(11)	1.406	1.397	−0.01

Table S3. *Cont.*

Bond Distances (Å)	Exp. (10a)	Cal. (10a) [a]	Dif.	Bond Distances (Å)	Exp. (10b)	Cal. (10b) [b]	Dif.
C(7)-H(5)	0.87	1.085	0.22	C(3)-C(7)	1.374	1.387	0.01
C(8)-C(10)	1.391	1.390	0.00	C(4)-C(6)	1.388	1.399	0.01
C(8)-C(11)	1.389	1.392	0.00	N(1)-C(6)	1.317	1.314	0.00
C(8)-H(10)	1.00	1.083	0.08	N(1)-C(7)	1.340	1.333	−0.01
C(9)-H(12)	0.95	1.083	0.13	C(5)-H(5)	0.931	1.084	0.15
C(9)-N(1)	1.333	1.333	0.00	C(5)-C(9)	1.394	1.390	0.00
C(10)-C(12)	1.382	1.397	0.01	C(5)-C(12)	1.387	1.392	0.01
C(10)-H(1)	0.92	1.085	0.13	C(7)-H(7)	0.930	1.083	0.15
C(11)-H(2)	1.03	1.083	0.05	C(8)-H(8)	0.930	1.084	0.15
C(13)-H(4)	0.91	1.091	0.18	C(8)-C(9)	1.374	1.392	0.02
C(13)-H(6)	0.94	1.091	0.15	C(9)-H(9)	0.930	1.083	0.15
C(13)-H(8)	1.07	1.093	0.02	C(10)-N(2)	1.484	1.477	−0.01
H(9)-N(2)	0.87	1.013	0.14	C(10)-C(11)	1.518	1.522	0.00
N(2)-S(1)	1.612	1.643	0.03	C(10)-H(10)	1.00	1.089	0.09
O(1)-S(1)	1.425	1.441	0.02	C(11)-C(12)	1.382	1.394	0.01
O(2)-S(1)	1.425	1.446	0.02	C(12)-H(12)	0.931	1.085	0.15

[a] Calculated geometry parameters for conformer **10a-1**; [b] Calculated geometry parameters for conformer **10b-1**.
